# Kiwifruit Information Resource (KIR): a comparative platform for kiwifruit genomics

**DOI:** 10.1093/database/bav113

**Published:** 2015-12-09

**Authors:** Junyang Yue, Jian Liu, Rongjun Ban, Wei Tang, Lin Deng, Zhangjun Fei, Yongsheng Liu

**Affiliations:** ^1^School of Biotechnology and Food Engineering, Hefei University of Technology, Hefei 230009, China,; ^2^School of Information Science and Technology, University of Science and Technology of China, Hefei 230009, China,; ^3^Information and Network Center, Hefei University of Technology, Hefei 230009, China,; ^4^Boyce Thompson Institute for Plant Research and USDA-ARS Robert W. Holley Center, Tower Road, Cornell University Campus, Ithaca, NY 14853, USA and; ^5^Ministry of Education Key Laboratory for Bio-Resource and Eco-Environment, College of Life Science and; ^6^State Key Laboratory of Hydraulics and Mountain River Engineering, Sichuan University, Chengdu 610064, China

## Abstract

The Kiwifruit Information Resource (KIR) is dedicated to maintain and integrate comprehensive datasets on genomics, functional genomics and transcriptomics of kiwifruit (*Actinidiaceae*). KIR serves as a central access point for existing/new genomic and genetic data. KIR also provides researchers with a variety of visualization and analysis tools. Current developments include the updated genome structure of *Actinidia chinensis* cv. Hongyang and its newest genome annotation, putative transcripts, gene expression, physical markers of genetic traits as well as relevant publications based on the latest genome assembly. Nine thousand five hundred and forty-seven new transcripts are detected and 21 132 old transcripts are changed. At the present release, the next-generation transcriptome sequencing data has been incorporated into gene models and splice variants. Protein–protein interactions are also identified based on experimentally determined orthologous interactions. Furthermore, the experimental results reported in peer-reviewed literature are manually extracted and integrated within a well-developed query page. In total, 122 identifications are currently associated, including commonly used gene names and symbols. All KIR datasets are helpful to facilitate a broad range of kiwifruit research topics and freely available to the research community.

**Database URL**: http://bdg.hfut.edu.cn/kir/index.html.

## Introduction

*Actinidia*, the basal genus within the *Actinidiaceae*, well known as kiwifruit, is widely distributed throughout most area of East Asia. The center for the origin of kiwifruit has been considered to be in the mountains and ranges around Southwestern China. Up to now, ∼54 species and 75 taxa have been described in *Actinidia* (1). Kiwifruit has a short history of domestication starting in the early 20th century when its seeds were introduced into New Zealand (2). Through decades of domestication and substantial efforts on selection from the wild kiwifruits, several economically important horticultural species have been developed, including *A.* *chinensis* Planchon, *Actinidiadeliciosa* (*A. chinensis* var. *deliciosa* A. Chevalier), *Actinidiaarguta* (Siebold and Zuccarini) Planchon ex Miquel and *Actinidiaeriantha* Bentham (3), all of which are perennial, deciduous and dioecious plants with climbing or straggling growth habit.

Nowadays, kiwifruit have become an important fresh fruit worldwide, due to its excellent nutritional qualities and unique flavor. Because of the remarkably high content of vitamin C and balanced nutritional composition of minerals, kiwifruit is commonly called ‘the King of fruits’ and ‘the King of vitamin C’. The flesh of the majority kiwifruit cultivars is either green, yellow or red when at harvest. Consumption of fresh kiwifruits has become populous around the world. The international kiwifruit industry, however, mainly relies on a few naturally selected cultivars from two infraspecific taxa of *A. chinensis* and *A. deliciosa* (4). The kiwifruit species are often reticulate polyploids with a basic chromosome number of *x* = 29 (5).

The extensive expressed sequenced tag (EST) sequences (6) provide valuable resources for investigating a wide variety of genetic characteristics of species, and facilitating the identification of candidate genes associated with agriculturally important traits as well as the understanding to important evolutionary processes. To date, > 130 000 ESTs have been deposited in the GenBank dbEST database (Table 1), and several high-density kiwifruit genetic maps have been published (7, 8). During the past several years, tremendous genomic and genetic data have been accumulated for kiwifruits.

**Table 1:** EST numbers of several kiwifruit species

*A. arguta*	7 259
*A.* *chinensis*	47 384
*A.* *deliciosa*	57 757
*A.* *eriantha*	12 650
*Actinidia hemsleyana*	5 101
*Actinidia indochinensis*	74
*Actinidia polygama*	1 348
*Actinidia setosa*	1 020

Early in 2011, the International Kiwifruit Genome Consortium (IKGC), initiated by scientists from Hefei University of Technology, Sichuan University and Cornell University, was dedicated to sequence the whole kiwifruit genome. One female individual of a Chinese kiwifruit cultivar ‘Hongyang’ (2n = 2x = 58) was selected for whole-genome sequencing project. In 2013, the sequencing was accomplished and the draft genome assembly along with its genome function was released at Kiwifruit Genome Database (http://bioinfo.bti.cornell.edu/cgi-bin/kiwi/home.cgi) (9). Depending on the initial version of the genomic sequence, IKGC has been committed to update the genome annotation and incorporate all available experimental data regarding gene structure and function (http://bdg.hfut.edu.cn/kir/news.html). Thus, there is an urgent need for a central platform to store, disseminate, mine and analyse these genomics and genetics datasets in time. For this end, we present the update kiwifruit database, Kiwifruit Information Resource (KIR), which currently contains the latest genome annotation, splice variants, predicted metabolic pathways, putative protein–protein interactions (PPIs) and associated research literature. A set of tools and user-friendly query interfaces have also been developed in the database to aid researchers in identifying and deciphering information of biological importance.

## Methods

### Gene prediction and annotation

A draft kiwifruit genome of 616.1 Mb was generated using the Illumina HiSeq 2000 system (9). The repeat-masked genome sequences identified by Repeatmasker (10) were used for gene prediction. Specifically, GeneScan (11) was employed for *de novo* gene prediction, while homologous sequence search was performed by comparison between the protein sequences of Arabidopsis, rice, grape and/or tomato using the TBLASTN program with an *E*-value cutoff 1*e*-5. EST sequences and transcriptome datasets were aligned to the repeat-masked kiwifruit genome sequences using BLAT (12). Finally, upon *ab initio* gene predictions and homologous sequence searching, integrated with the newest EST sequences and transcriptome of *A. chinensis*, a total of 39 761 protein-encoding genes were predicted. Among these genes, the average coding sequence length is 1123 bp and the average number of exon per gene is 8.6. Compared with the annotation of our first release, 9547 putative proteins are novel and 21 132 proteins are changed in coding sequences (e.g. alternative N terminal, alternative C terminal). Annotation of these predicted genes was performed by blasting against a number of nucleotide and protein sequence databases, including KEGG (13), non-redundant (nr) (14), Swiss-Prot (15), TrEMBL (16), TAIR (17), Solgenomics (18), Gene3D (19), Pfam (20), SUPFAM (21), HAMAP (22), PIRSF (23), PRINTS (24) and Prosite (25), using an *E*-value cutoff of 1*e*-5. Gene Ontology (GO) terms were assigned to the annotated genes in kiwifruit using the Blast2GO pipeline (26). To provide more information from the identifiers, we reassigned the identifiers for each gene and protein. Referring to the gene nomenclature system for Arabidopsis (17), tomato (18) and rice (27), the identifier for putative gene is designated as ‘AchXXgXXXXXX’. ‘Ach’ is the abbreviation of kiwifruit species name of *A.* *chinensis* in three characters. The two digits of a number following ‘Ach’ denote the chromosome, and the next letter ‘g’ means a putative gene. The last six digits of a number signify the accession number with counting in a single increment. Accordingly, proteins translated from the corresponding gene are named with a dot and a number following the gene name, where different number stands for different isoforms (e.g. AchXXgXXXXXX.1). Of these 39 761 annotated genes, 8245 contained two or more transcripts. Detailed information of the alternative variants is shown in the gene details view.

### Protein domain and transcription factor

Protein domains are conserved parts of proteins that recur throughout the protein world. They usually form compacted three-dimensional structures and function independently (28). In the predicted protein sequences of kiwifruit, conserved domains were identified by comparison against datasets from Pfam (20) and InterPro (29) databases. In total, 25 583 (∼64.3%) predicted proteins were annotated with one or more domains, and 3704 different domains were recorded. For the users’ convenience, we have collected and descripted 85 common domains, with a total of 1315 putative genes included. Zinc finger (Znf) domain has the largest number of members, followed by U-box and NAC domains. Among these putative genes with special domains, most are presumed to function as transcription factors (TFs) (30). TFs play a vital role in regulating gene expression by binding to specific DNA sequences and are involved in many key cellular processes. TFs were identified and classified into different families using the iTAK pipeline (http://bioinfo.bti.cornell.edu/tool/itak) (31). Integrated with the descriptions from http://planttfdb.cbi.pku.edu.cn (32), 1358 putative genes that have been classified into 52 TF families are listed in this section for an easy visualization. MYB gene family is the largest among all these predicted TF families, containing 203 gene members. The putative genes with identities of various protein domains and/or TF families are shown in a tabular format with links to their detailed information.

### Protein kinase and phosphatase

Protein phosphorylation and dephosphorylation play significant roles in almost all biological processes (33, 34). As one of the most important post-translational modifications of proteins, protein kinases (PKs) and protein phosphatases (PPs) are responsible for reversible phosphorylation (33). PKs are enzymes that phosphorylate target proteins by chemically adding phosphate groups to specific amino acid residues, whereas PPs catalyse the hydrolysis reaction of dephosphorylation through the removal of one or more phosphate groups from the substrates. Therefore, the identifications of PKs and PPs are fundamental for understanding the regulatory mechanisms of protein phosphorylation or dephosphorylation. Here, we used an online tool (http://ekpd.biocuckoo.org/advance.php) to predict potential PKs and PPs (35). As a result, 45 and 16 families of kinases and phosphatases are respectively annotated and listed in the web pages.

### Next-generation sequencing data

Currently, a large amount of high-throughput sequencing datasets are publicly available, which facilitates the precise comparison of gene sequences between different species and complements the genome annotation by mapping RNA-Seq datasets to the genome. The study of gene expression will deepen the understanding of gene/protein functions of kiwifruit. To illustrate the genome-wide gene expression profile derived from transcriptome datasets, we incorporated 18 publicly available illumina RNA-Seq datasets based on relative reads per million reads (RPKM) value. The RPKM value was calculated and normalized by referring to the aforementioned procedure and methods (9). These datasets include three kiwifruit species across three developmental stages of 20 days after pollination (DAP), 120 DAP and 127 DAP, which have been deposited in NCBI sequence read archive (SRA) under accession number SRA065642.

### Establishment of PPI

Based on the interactomes established with experimental verifications of Arabidopsis (*Arabidopsis thaliana*) (36), human (*Homo sapiens*) (37) and yeast (*Saccharomyces cerevisiae*) (38), we predicted the potential PPIs among proteins annotated from the kiwifruit genome datasets through orthologous mapping. The interactome datasets of Arabidopsis (18 462 pairs), human (151 226 pairs) and yeast (126 097 pairs) were downloaded from IntAct (15 June 2014 release, http://www.ebi.ac.uk/intact/) (39). We first detected orthologous clusters between kiwifruit and these three reference organisms using the BLAST program with an *E*-value cutoff of 1*e*-5. Subsequently, the orthologs were mapped onto the interactome datasets of the reference organisms. Any two proteins derived from kiwifruit with an orthologous interactive pair were recorded as an interacting protein group. As a result, 247 459 non-redundant PPIs among 6608 putative proteins, including 3495 homodimers (self-interactions) and 243 964 heterodimers (interacting between different proteins) PPIs, are obtained (which could be saved from the web site). In addition, the Cytoscape Web (40) is embedded within our web pages to display these identified PPI datasets.

### Literature-based manual curation

The above annotation has been implemented based on computational analyses and theoretical prediction. Although tremendous experimental data (e.g. protein sequences stored in Swiss Prot) and improved algorithm (e.g. BLAST algorithm) enabled us to predict highly reliable protein coding sequences and gene functions, manual curation of detailed information from the literature is extremely important for verifying the accuracy of the annotation. Therefore, we performed a literature-based manual curation to collect and organize update available information concerning the gene names, symbols, transcript and protein sequences, active substances, functional description and PMID. In addition to the information integrated into detailed description of genes/proteins, we have developed a standalone query page to facilitate user access (http://bdg.hfut.edu.cn/kir/advance.php). Seven categories are summarized: allergen detection, breeding improvement, disease resistant, flavor compounds, functional metabolites, human health and protein function. These literature-based annotation data will be updated regularly.

## Utility

### Database implementation

Currently, KIR operates on a Linux (Debian 7.8) system under an Apache HTTP server and uses MySQL as the database management system. PHP5, Perl scripts, HTML and JavaScript are employed to build the user-friendly interface and to write the web pages. Genome visualization is developed on JBrowse version 1.11.6 (41), which is a genome browser being developed as the successor to GBrowse. Online analysis tools in KIR are implemented as in-house PHP scripts or by wrapping existing third-party tools (e.g. BLAST server, Cytoscape Web).

### Query option

**Figure 1.**
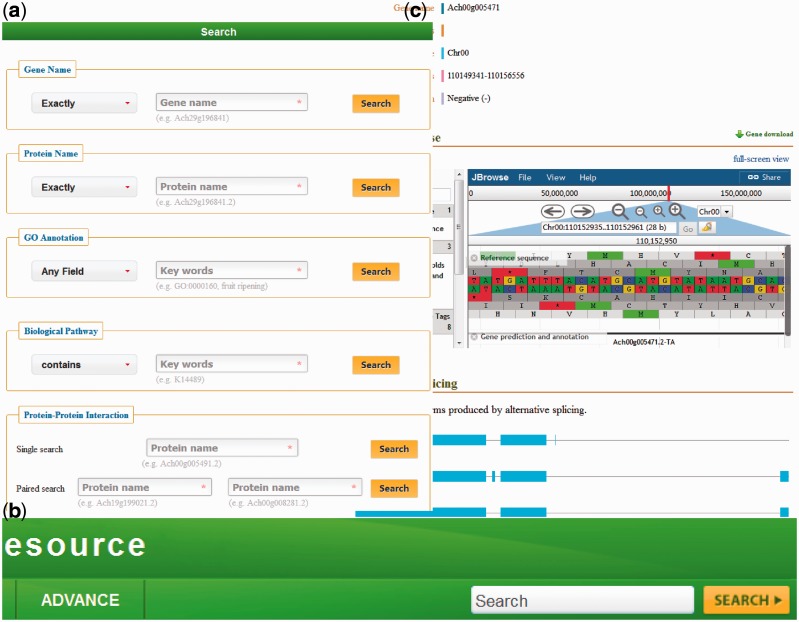
(**a**) The KIR data query interface; (**b**) the head search is provided at all pages; (**c**) the gene details view.

KIR is developed in an easy-to-use mode. To access the database contents, we provide five search categories: (i) with Gene Names, (ii) with Protein Name, (iii) with GO Annotation, (iv) with Biological Pathway and (V) with potential PPI (Figure 1a).

Query with gene/protein names is automatically normalized with synonyms. The full name and abbreviation are both feasible. Meanwhile, four optional conditions in the left drop-down box are provided, including ‘Exactly’, ‘Contains’, ‘Starts with’ and ‘Ends with’.

The third and fourth axes of query can be searched by various functional descriptions from GO annotation and Biological pathway. Both identifications and keywords are available. Users could generally find genes/proteins of interest with special characteristics.

The fifth search category contains two sub folders. Both ‘Single search’ and ‘Paired search’ are accession number centric. Users can find the potential PPIs with the identifiers of proteins. Only the record of two interested proteins will be found in the pair search folder.

In addition to the earlier search options, a head search is provided at all pages in the database (Figure 1b). This option could be very useful for a quick look up to the genes/proteins by searching either gene names or protein names.

### Detailed information

**Figure 2.**
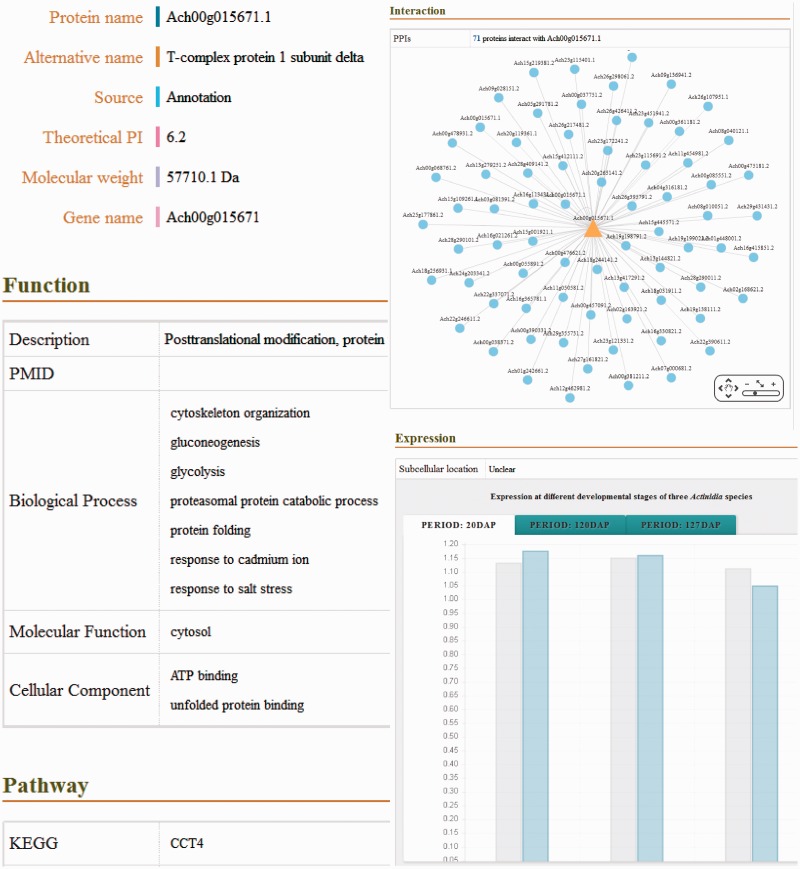
Screenshot of the detailed information of putative proteins, including basic information, functional description, biological pathway, molecular structure, predicted PPI, protein expression and related publications.

In the database, the detailed descriptions of individual genes and their corresponding coding proteins are displayed separately. The gene details view (Figure 1c) consists of four tabs: basic information including the gene names and symbols; the genome browse based on JBrowse; alternative splicing transcript deduced from transcriptome sequencing data and mutagenesis linking to PAAS database (42). The detailed information of proteins (Figure 2) is separated into ten tabs: basic information (e.g. name, alternative name, theoretical PI and molecular weight), function referring to manual curation and GO annotation (26, 43), pathway based on the KEGG analysis (13), interaction identified from orthologous interactions, sequences including the length as well as its isoforms and mutagenesis, similar protein (combination of the search records from database of GenBank, Swiss Prot, TrEMBL, Arabidopsis, Tomato and kiwifruit), expression information of the corresponding transcripts, protein structure with the secondary structure and three-dimensional structure annotation, family and domains (covering the annotations from Gene3D, Pfam, SUPFAM, HAMAP, PIRSF, PRINTS and Prosite database) and publications.

### Homology search

KIR offers a homology search function in BLAST, which is embedded in the database using the ncbi-wwwblast package (version 2.2.30) to provide a web-based graphical interface (44). Users can search homologous information by directly pasting query sequences in the text box. The whole draft genome sequence, putative gene sequences, coding sequences (CDS), protein sequences and EST datasets of eight kiwifruit species are organized as databases for BLAST search. Then, an appropriate search program (BLASTN, BLASTP, BLASTX, TBLASTN or TBLASTX) should be selected, where BLASTP and TBLASTN are only suitable for the query of amino acid sequences. Options for filtering low-complexity sequences and limiting the number of outputs are available as advanced options in the input boxes of ‘*E-*value’ and ‘Max. hits to show’, respectively. The format of output results could also be customized through the ‘Output options’ and ordered according to the expected value.

### Chromosome location

The chromosome location tool enables users to retrieve sequences of specific chromosomal region. By specifying any two linked positions, users can obtain the data between them, which would be helpful, e.g. to search for candidate genes of the target region detected by quantitative trait locus analysis or BLAST search. The retrieved data is shown directly in the window.

### Data downloads

Data files for genome sequences and structure information of kiwifruit are accessible from the ftp site via the ‘FTP Archive’ link in the main header. Fully annotated chromosome sequences are provided in Generic Feature Format 3 (GFF3) format, along with FASTA files of each chromosome sequence. Lists of data files for previous genome release are also available on the ftp site. In contrast to the ftp site that provided genome-wide datasets, a variety of gene functional description, transcriptome data and protein annotation files are provided in the form of flat-files. All data are hosted and accessible publicly. Users can also query for summarized information by gene/protein names, GO annotations, biological pathways and PPIs, which could be downloaded in comma-separated values format.

## Conclusion

KIR hosts a wide range of kiwifruit genomics and genetics datasets based on the latest and most accurate genome annotation. KIR aims to be a comprehensive online platform for users to search, visualize, download, retrieve literature and perform cross-species queries with unrestricted public access. It integrated all publicly available high-throughput sequences, experimental datasets, links to other useful databases and related analysis tools. To provide up to date gene/protein annotations, we will continue to collect and incorporate literature-based curation data. We believe that KIR will be a useful database for researchers and breeders who are working on kiwifruit.
